# Apolipoprotein E knockout rabbit model of intracranial atherosclerotic disease

**DOI:** 10.1002/ame2.12125

**Published:** 2020-06-21

**Authors:** Matthew S. Zabriskie, Chuanzhuo Wang, Shuping Wang, Matthew D. Alexander

**Affiliations:** ^1^ Department of Radiology and Imaging Sciences University of Utah Salt Lake City UT USA; ^2^ Department of Radiology Shengjing Hospital of China Medical University Shenyang China; ^3^ Department of Neurosurgery University of Utah Salt Lake City UT USA

**Keywords:** animal model, histopathology, intracranial atherosclerosis, ischemic stroke, vessel biology

## Abstract

Intracranial atherosclerotic disease (ICAD) is the most common cause of ischemic stroke. Poor understanding of the disease due to limited human data leads to imprecise treatment. Apolipoprotein E knockout (ApoE‐KO) rabbits were compared to an existing model, the Watanabe heritable hyperlipidemic (WHHL) rabbit, and wild‐type New Zealand white (NZW) rabbit controls. Intracranial artery samples were assessed on histopathology for the presence of ICAD. Logistic and ordinal regression analyses were performed to assess for disease presence and severity, respectively. Eighteen rabbits and 54 artery segments were analyzed. Univariate logistic analysis confirmed the presence of ICAD in model rabbits (*P* < .001), while no difference was found between WHHL and ApoE‐KO rabbits (*P* = .178). In multivariate analysis, only classification as a model vs wild‐type animal (*P* < .001) was associated with the presence of ICAD. Univariate ordinal regression analysis demonstrated an association between ICAD severity and model animals (*P* = .001), with no difference was noted between WHHL and ApoE‐KO rabbits (*P* = .528). In multivariate ordinal regression analysis, only classification as a model retained significance (*P* < .001). ICAD can be reliably produced in ApoE‐KO rabbits, developing the disease comparably to the older WHHL model. Further analysis is warranted to optimize accelerated development of ICAD in ApoE‐KO rabbits to more efficiently study this disease.

## 1 INTRODUCTION

Intracranial atherosclerotic disease (ICAD) is the most common cause of stroke worldwide.^1^, ^2^, ^3^ Pathophysiological mechanisms of ICAD have not been clarified in a way that is specific to intracranial disease, so current clinical management strategies are suboptimal. Better understanding of ICAD is needed through basic science and translational research to develop more effective treatment. A major hindrance in the study of ICAD pathophysiology is the lack of tissue for pathological evaluation, but this cannot be reliably obtained in humans. Biopsy is prohibitively morbid, so tissue is typically only obtained at autopsy.

Given limitations in human investigation of ICAD, current management is based on extrapolation from extracranial disease. Such extrapolation may be flawed since intracranial and extracranial vessels arise from different germ cell layers, ectoderm, and mesoderm, respectively, so it should not be assumed that these vessels and the diseases they may harbor are the same.^4^, ^5^, ^6^ Several differences are known between intracranial and extracranial arteries, such as the presence or absence of vasa vasorum, size of adventitia, response to systemic processes like serum cholesterol or hypertensions, and their differential involvement in genetic disorders like familial hypercholesterolemia.^4^, ^5^, ^7^, ^8^, ^9^, ^10^, ^11^, ^12^ Further evaluation of ICAD is needed, including comparisons to extracranial disease that test the appropriateness of the current extrapolation from extracranial to intracranial vessels. Given the paucity of human data, an animal model is critically needed.

Animal models for the study of atherosclerosis have been reported in species as small as mice and as large as nonhuman primates, with most work occurring in rodent and rabbit models.^13^, ^14^ Apolipoprotein E knockout (ApoE‐KO) mice have been shown to develop intracranial atherosclerosis; this approach is based on understanding of the plasma lipoprotein's role in cholesterol degradation and association with premature atherosclerosis in humans when deranged.^15^, ^16^ While useful for certain investigations, large animal models are necessary for better assessment with noninvasive imaging and surgical procedures that translate to human care.^14^, ^17^ As in humans, most rabbit atherosclerosis research involves extracranial vessels. The most widely studied rabbit model of atherosclerosis is the Watanabe heritable hyperlipidemic (WHHL) rabbit.^14^, ^18^, ^19^, ^20^ WHHL rabbits develop ICAD, although some evidence exists that it typically only occurs after the induction of hypertension.^21^, ^22^ To date, most WHHL investigation has focused on extracranial disease, and the mutation underlying this strain is not associated with intracranial disease in humans.^14^, ^19^, ^20^, ^21^, ^22^ Additionally, in recent years, steady supply of these animals has not been maintained, so a better model of ICAD is needed.^23^ Apolipoprotein E knockout (ApoE‐KO) rabbits have been used for the evaluation of extracranial atherosclerosis, but they have not been evaluated for the presence of ICAD.^24^ This study describes the comparisons of intracranial arteries in WHHL and ApoE‐KO rabbits with comparison to NZW controls.

## 2 METHODS

All studies were conducted in compliance with a protocol approved by the institutional animal care and use committee. Mature WHHL and ApoE‐KO rabbits fed a regular or custom atherogenic diet (0.3% cholesterol, 3% soy bean oil, Envigo Teklad Diets), as well as younger NZW rabbits, were also analyzed.^23^ WHHL rabbits were sourced from the Shiomi Laboratory at Kobe University. ApoE‐KO rabbits were provided by Horizon Discovery Group, which was subsequently acquired by Envigo, Indianapolis, IN, who also supplied NZW rabbits.

For euthanasia under general endotracheal anesthesia induced and maintaining with isoflurane, a midline neck incision was made over the trachea. The right carotid artery was isolated using blunt dissection; gentle tension was applied with vessel loops. A 22‐gauge intravenous catheter was inserted into the artery. A 5‐French micropuncture sheath was then placed in the artery over a 0.018 in microwire using Seldinger technique. This sheath was secured within the vessel with a 2‐0 silk suture. After removing the inner dilator and wire, the sheath was connected to the perfusion pump to deliver 2% paraformaldehyde and 5% glutaraldehyde solution. After initiating perfusion, a jugular vein was transected to limit blood in the vessels and tissues after exsanguination. Following adequate perfusion, the animal was decapitated to harvest the brain and its intact arteries, which were placed in formalin.

After at least 2 weeks in formalin, tissue was sliced to maximize cross‐sectional orientation of basilar and internal carotid arteries. Hematoxylin and eosin staining was performed and slides were created and studied with light microscopy. Internal carotid and basilar artery segments were each rated as having no, mild, moderate, or advanced ICAD. Severity was determined by histological findings including smooth muscle hypertrophy, wall thickening, infiltration of inflammatory cells, neointimal formation, presence of lipids within neointima, and remodeling of the vessel lumen.

Descriptive statistics were performed, assessing disease burden on a by‐segment basis. Further analysis was performed to compare animal features and disease burden. When assessing for the presence of disease, binary logistic regression analysis was performed. When assessing for severity of disease, ordinal regression analysis was performed. Multivariate models were constructed, including variables with *P* < .010 in univariate analysis, to assess for confounding factors. Statistics were performed using R (R Foundation for Statistical Computing, Vienna, Austria).

## 3 RESULTS AND DISCUSSION

**Figure 1:**
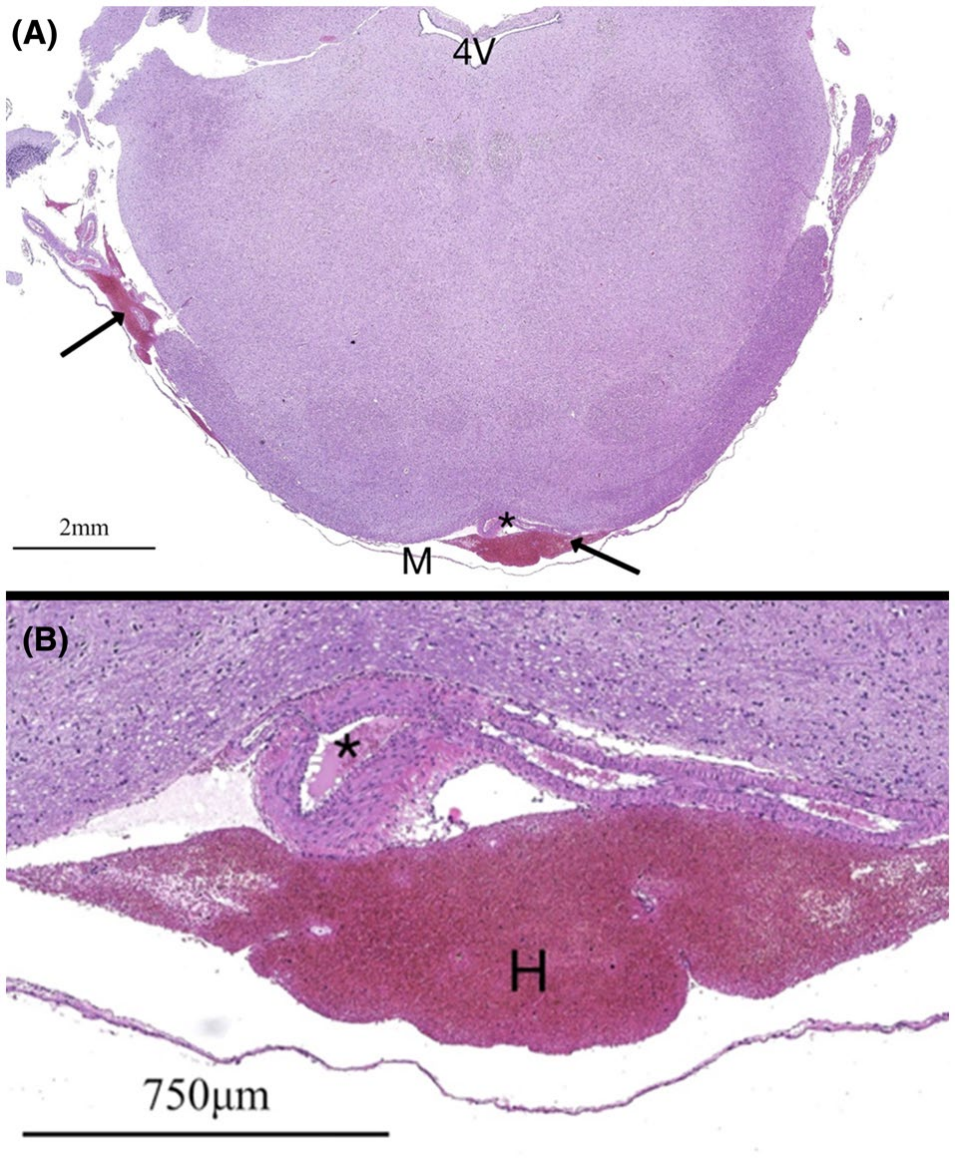
H&E‐stained specimens from a NZW rabbit (animal 13) at the level of the brainstem and fourth ventricle (4V) at 10× (A) and 200× (B) magnification. A decompressed normal basilar artery is noted (*), the lumen of which contains erythrocytes from poor clearance during perfusion. Subleptomeningeal hemorrhage (H, arrows) is noted from complications during fixation

**Figure 2:**
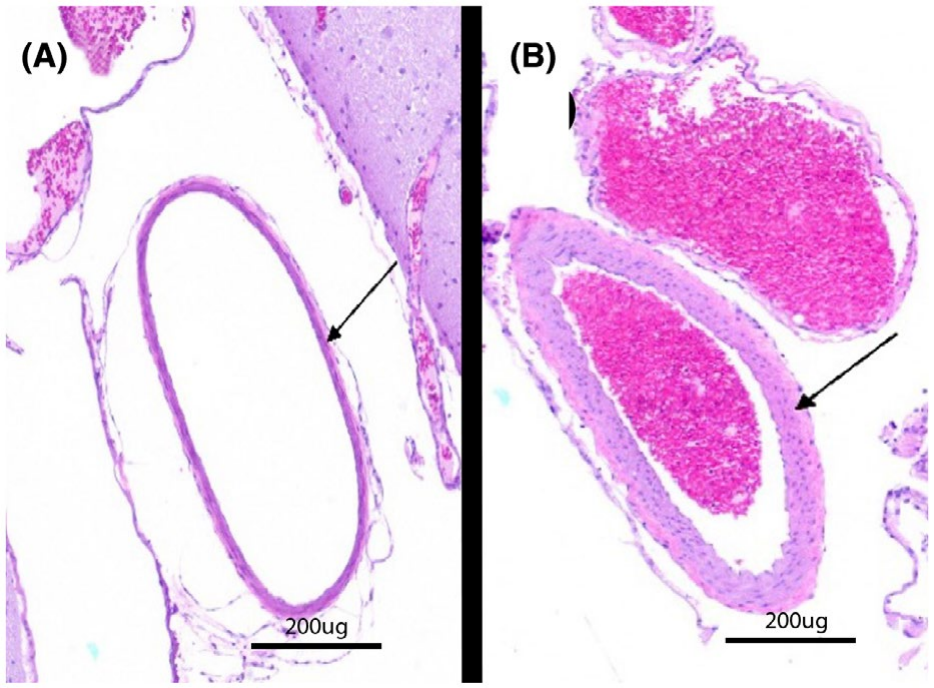
H&E‐stained specimens of left ICAs from two different ApoE‐KO rabbits with thin but normal (A, animal 6) and hypertrophied (B, animal 10) arterial walls, the latter of which is consistent with mild atherosclerotic changes

**Figure 3:**
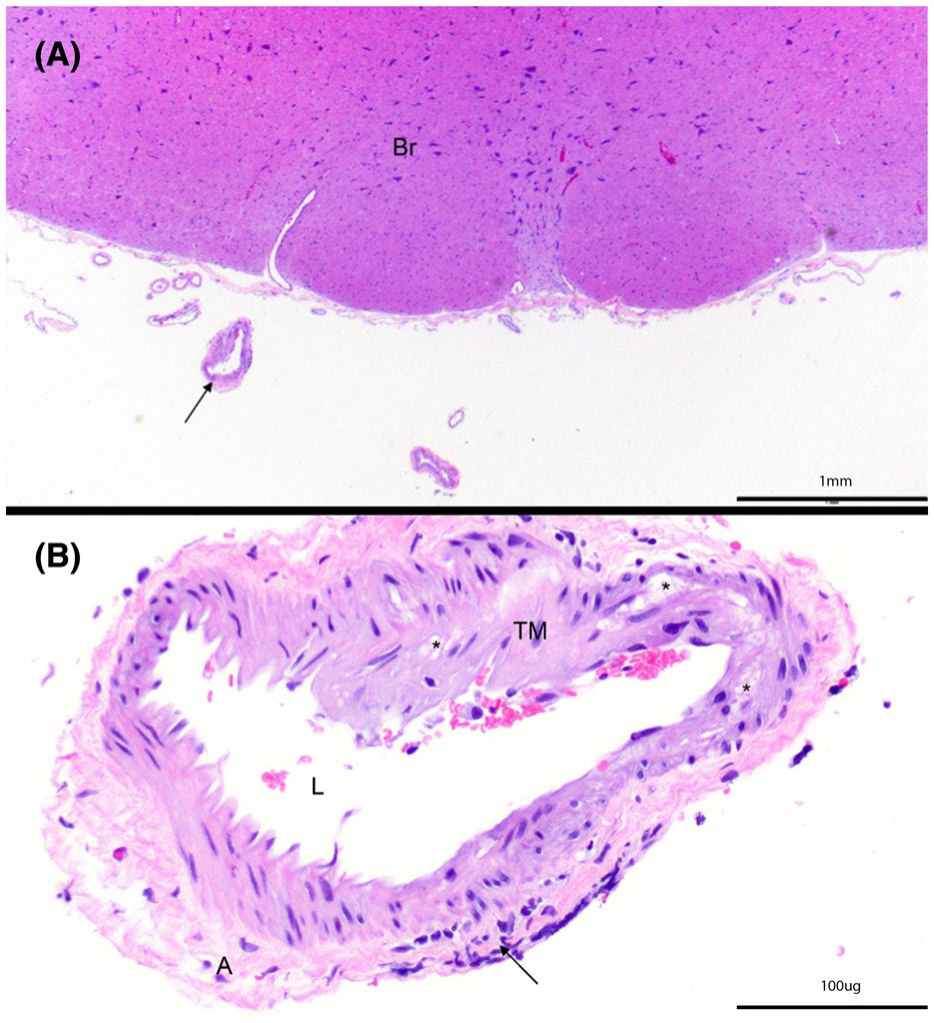
H&E‐stained specimen from an ApoE‐KO rabbit (animal 8) at (A) 20× and (B) 200× at the level of the brainstem (Br) demonstrate expansion of the tunica media (TM) by vacuolated cells (*). Black arrow indicates inflammatory cell infiltration. Adventitia (A) and lumen (L) are also indicated. Note, image B is rotated clockwise compared to image A. Arterial findings are consistent with moderate atherosclerosis

**Figure 4:**
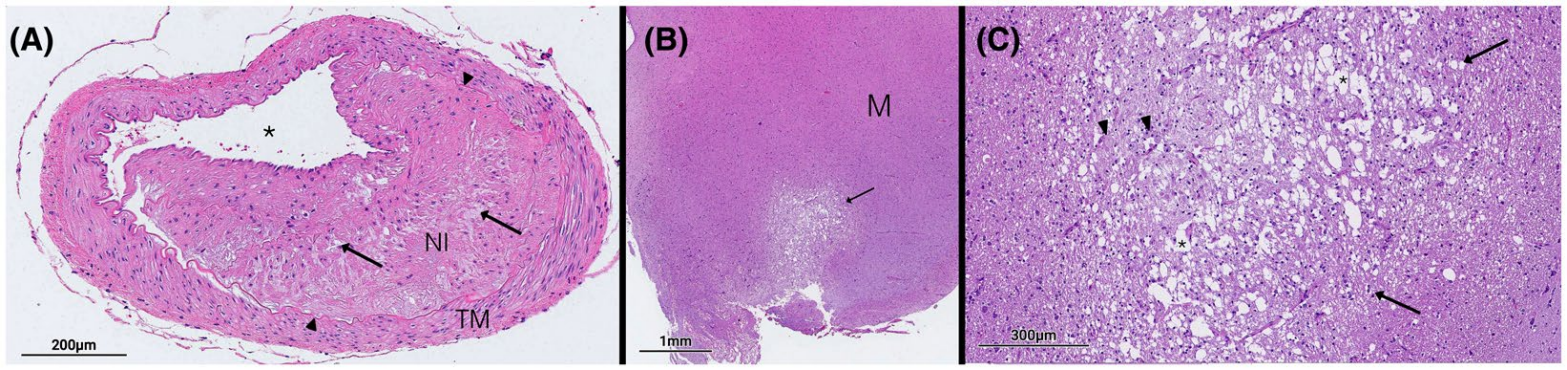
H&E‐stained specimens through the (A) basilar artery and (B, C) midbrain of a WHHL rabbit (animal 3). The basilar artery demonstrates arterial wall hypertrophy, pronounced neointimal formation (NI) demarcated by black arrows, hypetrophy of the tunica media (TM), and lipid deposition within the atheroma, all consistent with an advanced lesion. (B, C) Rarefaction through the midbrain demonstrates an area of infarct due to the atherosclerotic plaque

**Figure 5:**
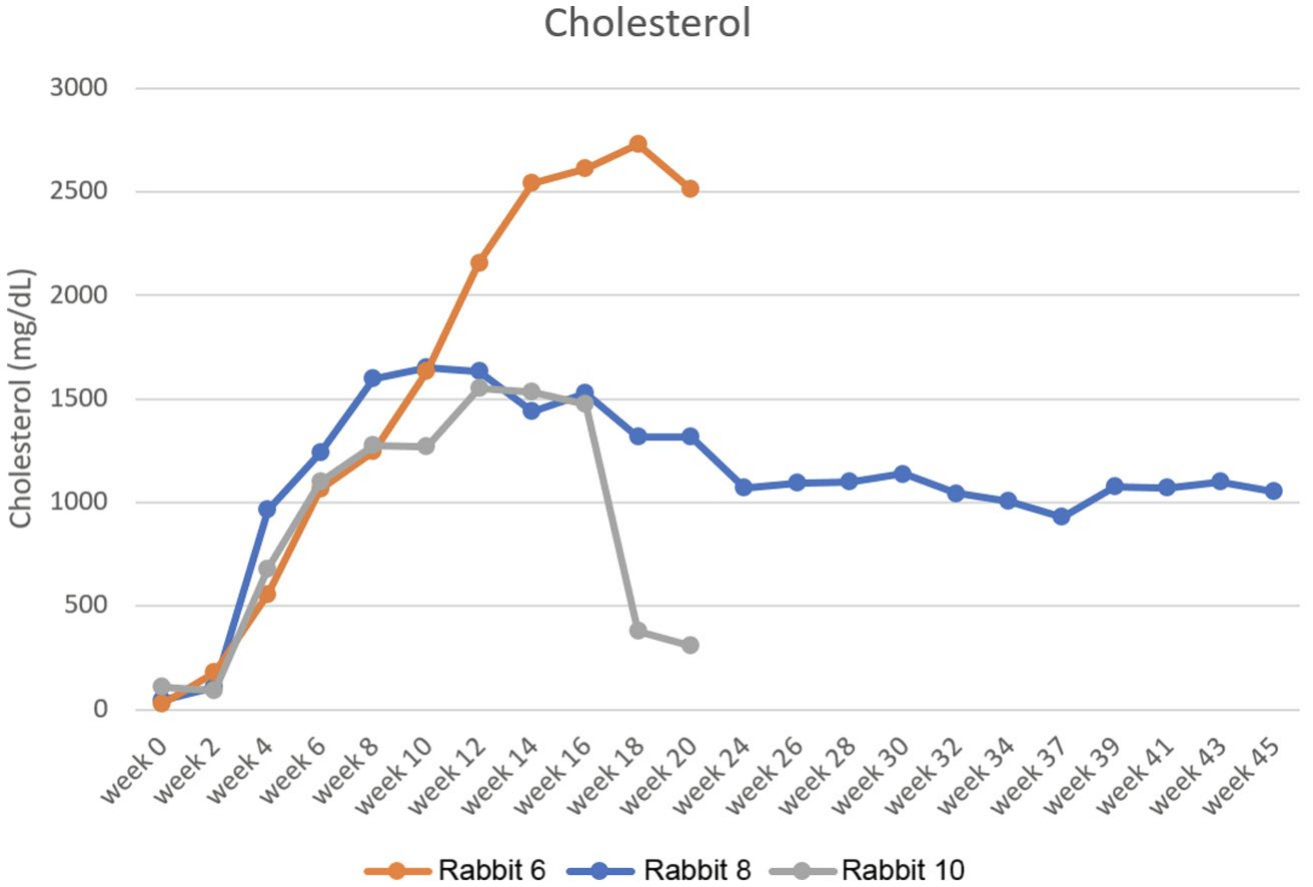
Plots of serum cholesterol levels in three ApoE‐KO rabbits (animals 6, 8, and 10 in Table 1). Weeks on the x‐axis start on at the initiation of custom diet. Of note, animal 10 began refusing custom feed pellets around week 16 and was subsequently switched to a normal diet

Eighteen rabbits (5 WHHL, 5 ApoE‐KO, and 8 NZW) and 54 artery segments underwent evaluation. Table 1 lists animal features and histopathology results for each segment. Representative images of various ICAD lesion severities are provided in Figures 1, 2, 3, 4. Univariate analysis confirmed the presence of ICAD in model rabbits compared to NZW rabbits (*P* < .001), with no difference between WHHL and ApoE‐KO rabbits (*P* = .178). ICAD was more commonly found in older (*P* = .003) and heavier (*P* = .001) rabbits and those fed a custom diet (*P* = .023). No association was noted for vessel segment (*P* = .127). A nonsignificant trend was noted for more disease occurrence in the posterior vs anterior circulation (0.079). In multivariate analysis assessing the presence of ICAD, only classification as a model (*P* < .001) was associated with the presence of ICAD. Figure 5 plots serum cholesterol results from animals 6, 8, and 10, which were tracked to observe response to custom diet.

**Table 1:** Rabbit features and pathology findings

Animal	Strain	Age(mos)	Sex	Mass(kg)	Diet	ICAD severity on pathology		
						Right ICA	Left ICA	Basilar
1	WHHL	28.0	M	2.66	Regular	None	None	Mild
2	WHHL	31.7	F	3.41	Regular	None	None	Moderate
3	WHHL	24.0	M	2.68	Regular	None	None	Advanced
4	WHHL	34.4	F	3.31	Regular	None	None	None
5	WHHL	25.6	M	3.8	Regular	Mild	None	Moderate
6	ApoE	47.0	M	4.4	5.0 mos, Custom	None	None	None
7	ApoE	38.3	M	4.79	0.9 mos, Custom	Moderate	None	None
8	ApoE	52.0	F	3.56	10.8 mos, Custom	None	Mild	Moderate
9	ApoE	36.3	M	4.8	Regular	None	Mild	Mild
10	ApoE	46.1	M	4.33	4.9 mos, Custom	Mild	Mild	Mild
11	NZW	8.1	F	3.49	Regular	None	None	None
12	NZW	10.6	F	3.37	Regular	None	None	None
13	NZW	7.1	F	4.28	Regular	None	None	None
14	NZW	21.5	F	2.9	Regular	None	None	None
15	NZW	9.9	F	3.56	Regular	None	None	None
16	NZW	7.7	F	3.59	Regular	None	None	None
17	NZW	7.7	F	2.4	Regular	None	None	None
18	NZW	27.6	F	4.33	Regular	None	None	None

Univariate ordinal regression analysis demonstrated an association between ICAD severity and model animals (*P* = .001), with no difference between WHHL and ApoE‐KO rabbits (*P* = .528). Severe ICAD was more common with advanced age (*P* = .056), greater mass (*P* = .016), and among those fed a custom diet (*P* = .005). More severe disease was noted in the posterior circulation (*P* = .045), although no association was found when looking at individual segments (*P* = .095). In a multivariate ordinal regression analysis, only classification as a model retained significance (*P* < .001).

Current ICAD management is based on extrapolation from extracranial disease in which systemic processes lead to lesion development at vulnerable locations like bifurcations points or bends in arteries, but little research has actually studied the intracranial vessels themselves.^25^, ^26^ Different germ cell layers give rise to intracranial and extracranial arteries.^4^, ^5^, ^6^ If assumptions about ICAD can be made based on the understanding of extracranial disease, it must first be demonstrated that intracranial and extracranial vessels and diseases are in fact similar. Very little study has investigated differences between ICAD and extracranial atherosclerosis, yet management of ICAD still reflects extracranial disease management. There is acute need for better pathophysiological data on ICAD, but human investigations alone will not be sufficient. A robust animal model is sorely needed.

Most of the existing work on intracranial disease has occurred in WHHL rabbits. These animals lack low‐density lipoprotein receptors, which in turn leads to the development of atherosclerosis.^14^, ^18^, ^19^, ^27^, ^28^ Research on WHHL rabbits led to the discovery of the underlying mutation causing familial hypercholesterolemia.^19^ ICAD typically does not occur in patients with familial hypercholesterolemia, and the overwhelming majority of ICAD patients do not suffer from this rare disease, so WHHL rabbits may not be an appropriate ICAD model.^29^, ^30^, ^31^ Additionally, WHHL rabbits have poor general health. ICAD is reported to occur most reliably only after hypertension induction; this may further complicate the health of these fragile animals and prevent reliable long‐term analysis.^21^, ^22^

Other methods of atherosclerosis induction exist for rabbits.^14^ ApoE is involved in lipid transport and has been targeted for disease models in multiple species; knockout rabbits can now be selectively bred.^24^, ^32^, ^33^ Our research sought to assess ApoE‐KO rabbits as a model of ICAD, comparing them to WHHL rabbits and NZW controls. Based on previously reported methods and with the aim to accentuate and accelerate any ICAD development, most ApoE animals were fed moderately atherogenic diets during exploratory investigation to find a replacement for WHHL rabbits when they became unavailable.^14^, ^34^, ^35^, ^36^, ^37^ Variability in exposure to the custom diet resulted from different timeframes between acquisition and euthanasia, and some animals refused to eat the custom pellets and had to be switched to standard feed. Both WHHL and ApoE‐KO rabbits were found to have ICAD, while no NZW control animals harbored the disease. It is notable that WHHL animals reliably developed the disease without hypertension induction.

Several limitations of this preliminary analysis warrant discussion. Most ApoE‐KO animals received an atherogenic diet, whereas WHHL and NZW counterparts received regular diets. Due to logistical issues, NZW control animals were notably younger; age implications should be explored further in future research. Currently, it can reasonably be expected that disease only occurs late in the animal's life, so methods should be sought to accelerate the course of the disease to make efficient evaluation of the entire natural history more practical. In total, ApoE‐KO rabbits likely represent the most suitable ICAD model moving forward. Further robust investigation of ApoE‐KO rabbits to model ICAD is critically needed to lead to more effective treatments for this debilitating disease. This model can help shed light on recently described processes affecting extracranial atherosclerosis like inflammation, transcriptomic features, noncoding RNA, and neutrophil extracellular traps, specifically investigating the intracranial circulation to develop the next generation of diagnostic and therapeutic tools.

## CONFLICT OF INTEREST

None.

## AUTHOR CONTRIBUTIONS

All the authors participated in animal care, including perfusion fixation. MDA performed statistical analysis and authored the manuscript. Other authors edited the manuscript and approved the final version.
